# First person – Arun-Kumar Kaliya-Perumal

**DOI:** 10.1242/dmm.050846

**Published:** 2024-05-31

## Abstract

First Person is a series of interviews with the first authors of a selection of papers published in Disease Models & Mechanisms, helping researchers promote themselves alongside their papers. Arun-Kumar Kaliya-Perumal is first author on ‘Genetic regulation of injury-induced heterotopic ossification in adult zebrafish’, published in DMM. Arun conducted the research described in this article while a PhD student in Prof. Philip Ingham’s lab at Lee Kong Chian School of Medicine, Nanyang Technological University, Singapore. He will soon join the Rehabilitation Research Institute of Singapore as a Research Fellow, where he will pursue his interest in translational research within the field of orthopedics, focusing on enhancing the quality of healthcare delivery in Singapore.


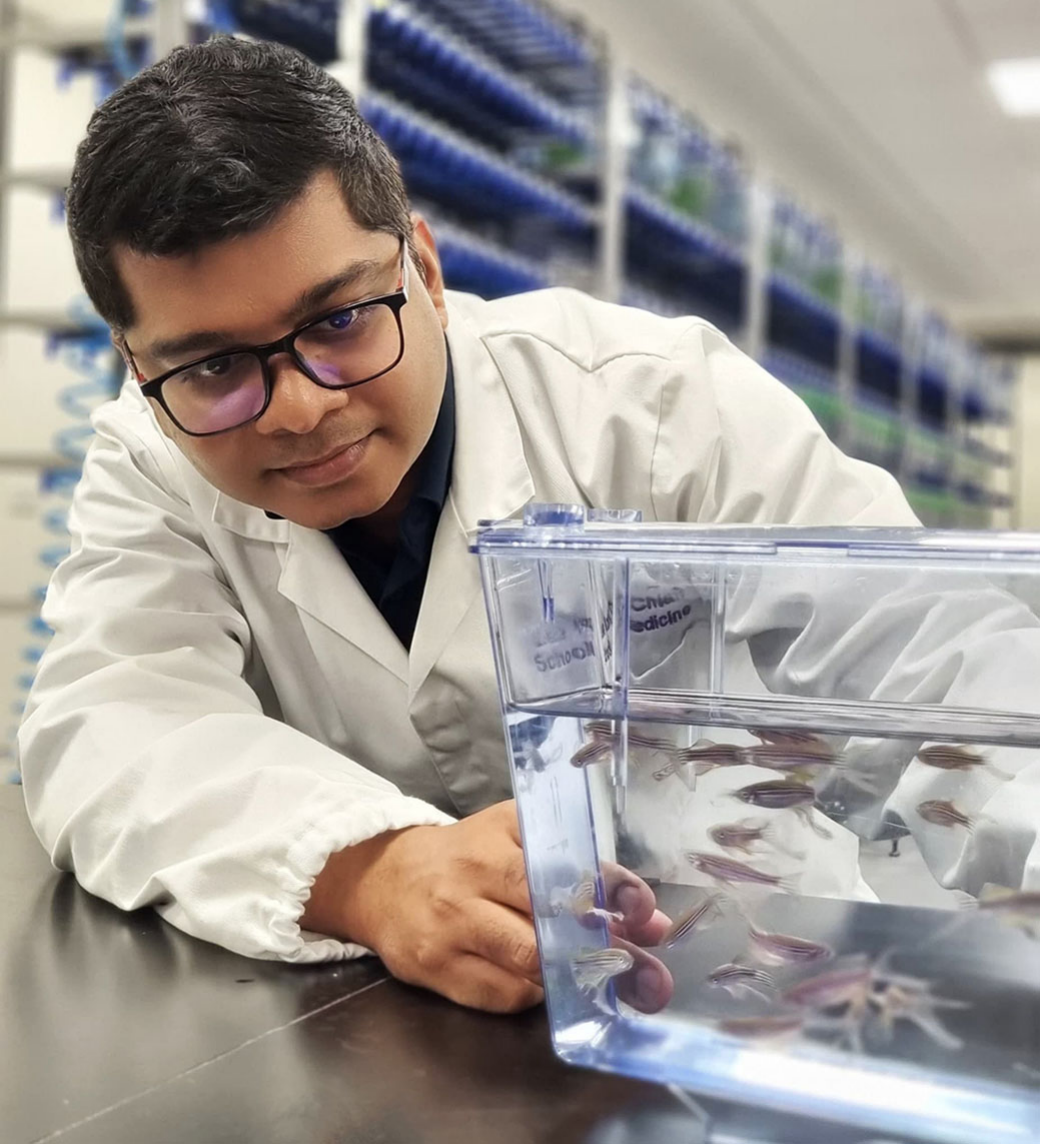


**Arun-Kumar Kaliya-Perumal**

**Who or what inspired you to become a scientist?**

Throughout my career as a clinician, specializing in orthopaedic and spine surgery, I have harboured a keen interest in research. While predominantly engaged in clinical research with patients, it was my experiences under mentors in Taiwan during my clinical fellowship in spine surgery that truly inspired me. Working with them in both basic sciences and clinical research, I realized the importance of clinician scientists being at the intersection between the two realms. With this realization in mind, following a research-intense advanced spine surgery fellowship in Singapore, I made the decision to transition to full-time research, embarking on a PhD focused on unravelling the complexities of injury-induced heterotopic ossification, a condition that I used to treat in my home country of India.

**What is the main question or challenge in disease biology you are addressing in this paper? How did you go about investigating your question or challenge?**

The main challenge to finding a cure for injury-induced heterotopic ossification is the limited number of dependable models and the complexity of establishing them, which hinders large-scale drug testing. However, recognizing the striking parallels between zebrafish and mammals in musculoskeletal development and remodelling, we employed zebrafish to establish a simple and reliable model, enabling further exploration of the underlying mechanisms by RNA sequencing. Additionally, in collaboration with Prof. Matthew Harris from the Department of Genetics at Harvard Medical School in Boston, who offered me a 3-month research attachment in his lab, I was able to work on mutants that showed altered heterotopic bone formation response, highlighting the utility of the model that we established.

**How would you explain the main findings of your paper to non-scientific family and friends?**

One of the rare complications following musculoskeletal injury is the formation of ectopic bone in soft tissue sites. When such bone forms, particularly adjacent to joints, it restricts movement. Our research has established a zebrafish model that mimics this human condition. Using this model, we investigated the underlying changes in detail and discovered a few genes that could potentially drive this phenomenon. Furthermore, in a couple of mutant fish, we observed varying ectopic bone formation responses following injury, unlike what we observed in wild-type fish. These findings take us a step closer to finding a solution for mitigating heterotopic ossification.

The ability to consistently induce changes in bone structure following minor injuries […] establishes the zebrafish as a potent and reliable experimental model for further research into heterotopic ossification.

**What are the potential implications of these results for disease biology and the possible impact on patients?**

Our investigation unveils for the first time in zebrafish, the occurrence of injury-induced heterotopic ossification. The ability to consistently induce changes in bone structure following minor injuries, alongside the available imaging options, firmly establishes the zebrafish as a potent and reliable experimental model for further research into heterotopic ossification. As a proof of concept, we have also demonstrated varied heterotopic bone formation responses in mutant fish. With further validation, the potential therapeutic targets identified here could pave the way for developing medical management strategies to treat human patients grappling with this debilitating condition.

**Figure:**
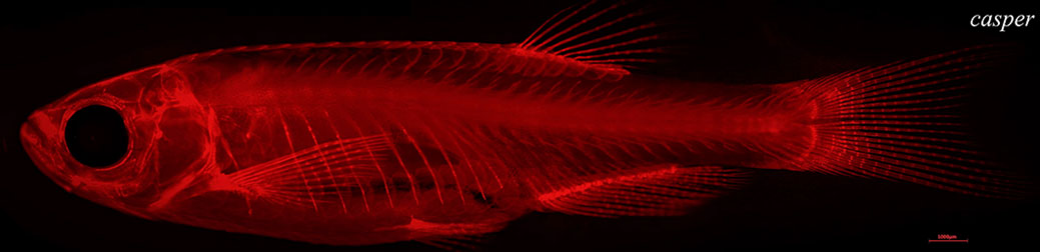
**Live Alizarin Red-stained *casper* mutant zebrafish showing the entire ossified skeleton in bright red fluorescence.** Scale bar: 1000 μm.

**Why did you choose DMM for your paper?**

I personally had DMM in mind since we first published a review in 2020 on fibrodysplasia ossificans progressiva, under the Clinical Puzzles section (Kaliya-Perumal et al., 2020). Moreover, DMM is an ideal fit for our research and a deliberate selection, as the journal's scope perfectly aligns with our main aims of establishing a new model and leveraging it to unravel the underlying mechanisms. Aligning with our previous experience, the review process was seamless, ensuring highest scientific standards. As we continue our scientific journey, we remain indebted to DMM for providing us with a home for our scientific narratives.

**Given your current role, what challenges do you face and what changes could improve the professional lives of other scientists in this role?**

Resource constraints, such as limited access to equipment or funding, can significantly impede experiments and research progress. Overcoming this challenge often just means getting the right help to find alternative resources. Regrettably, not all early-career researchers receive such support. In such circumstances, collaborations can emerge as a crucial solution. Personally, I experienced the advantages of collaboration during my visit to Prof. Matthew Harris' lab in Boston. There, I had the opportunity to explore specific mutants of interest and conduct three-dimensional reconstructed micro-computed tomography scans for volumetric analysis of zebrafish bone. This collaboration provided access to resources and expertise that were otherwise unavailable to me.

**What's next for you?**

I am embarking on an exciting journey as a Research Fellow at the Rehabilitation Research Institute of Singapore (RRIS), a new collaborative institute between Nanyang Technological University (NTU), the Agency for Science, Technology and Research (A*STAR), and the National Healthcare Group (NHG) in Singapore. Here, my focus will be on interdisciplinary research in the field of musculoskeletal rehabilitation to enhance the quality of healthcare delivery in Singapore. Meanwhile, I will seek out funding opportunities to further explore the intriguing findings that emerged from my PhD project.

**Tell us something interesting about yourself that wouldn't be on your CV**

Whether it's watching adorable cat videos online or simply visiting cat cafes for a meal, my love for cats brings a sense of warmth and happiness to my life outside of work. I absolutely adore these graceful and independent creatures. Back in India, I have a British Longhair cat named Oreo, who thinks I'm just occupying his house. I love observing his stubborn yet endearing behaviour and cherish the time I spend with him. In Singapore, we have community cats that reside in public spaces across the country. People take care of these cats by providing timely food and necessary medical attention. I'm happy to contribute to their care with what is possible from my side. It's a wonderful feeling.
